# *QuickStats:* Reason for the Most Recent Colonoscopy,* Among Adults Aged 50–75 Years Who Had a Test in the Past 10 Years — National Health Interview Survey,† United States, 2018

**DOI:** 10.15585/mmwr.mm6924a5

**Published:** 2020-06-19

**Figure:**
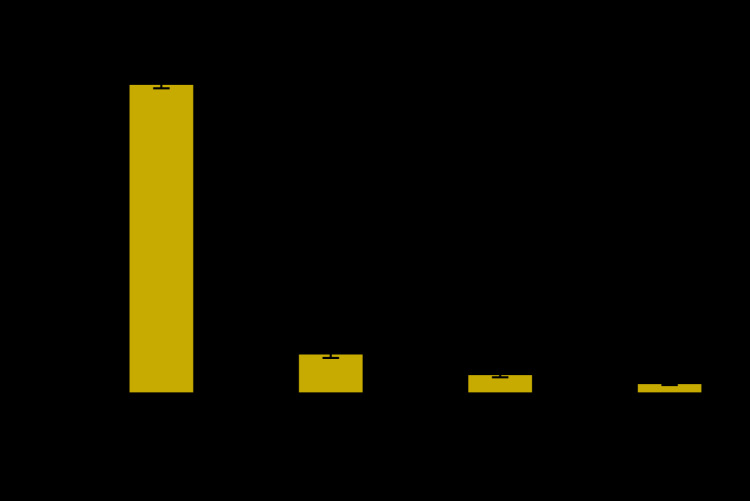
In 2018, 60.6% of U.S. adults aged 50–75 years without a personal history of colorectal cancer had a colonoscopy in the past 10 years. Of these, 81.2% had their most recent colonoscopy as part of routine screening, 10.6% had their most recent colonoscopy because of a problem, 5.2% as a follow-up to an earlier test or screening exam, and 2.8% for some other reason.

**Source:** National Health Interview Survey, 2018. https://www.cdc.gov/nchs/nhis.htm.

